# Early warning signals for critical transitions in a thermoacoustic system

**DOI:** 10.1038/srep35310

**Published:** 2016-10-21

**Authors:** E. A. Gopalakrishnan, Yogita Sharma, Tony John, Partha Sharathi Dutta, R. I. Sujith

**Affiliations:** 1 Department of Aerospace Engineering, Indian Institute of Technology Madras, 600036, India; 2 Department of Mathematics, Indian Institute of Technology Ropar, 140001, India

## Abstract

Dynamical systems can undergo critical transitions where the system suddenly shifts from one stable state to another at a critical threshold called the tipping point. The decrease in recovery rate to equilibrium (critical slowing down) as the system approaches the tipping point can be used to identify the proximity to a critical transition. Several measures have been adopted to provide early indications of critical transitions that happen in a variety of complex systems. In this study, we use early warning indicators to predict subcritical Hopf bifurcation occurring in a thermoacoustic system by analyzing the observables from experiments and from a theoretical model. We find that the early warning measures perform as robust indicators in the presence and absence of external noise. Thus, we illustrate the applicability of these indicators in an engineering system depicting critical transitions.

Many complex systems such as ecosystems, climate systems, financial markets and neurons in the mammalian cortex exhibit critical transition^1^,^2^,^3^,^4^,^5^,^6^,^7^,^8^,^9^,^10^. In first order transitions, the transition occurs at the bifurcation points (the so called tipping points) where the system abruptly shifts from one stable state to another stable state. This state shift can have undesirable consequences^11^ ranging from the extinction of species in ecosystems^12^,^13^ to sudden crash of economy in financial markets^14^. The undesirable state following a critical transition creates the need to develop early warning measures to detect the proximity of the system under consideration to a critical point or tipping point. Early detection of critical transitions has great relevance because it can initiate appropriate management strategies to prevent a forthcoming catastrophe^15^.

Over the years, many early warning measures have been developed to successfully detect critical transitions observed in complex systems. These early warning measures perform by exploiting the phenomenon of critical slowing down observed in dynamical systems. Dynamical systems display slow recovery from perturbations in the vicinity of a tipping point which is known as critical slowing down^16^,^17^. The signature of critical slowing down can be observed as an increase in variance, lag-1 autocorrelation and conditional heteroskedasticity prior to a critical transition^12^,^14^,^18^. Scheffer *et al*.^14^, in their pioneering study, proposed a plethora of statistical quantities such as recovery rate, variance and autocorrelation to forewarn imminent shifts in ecosystems, collapse of financial markets, and the onset of asthmatic attacks and epileptic seizures. Thereafter, these early warning measures are extensively used to predict critical transitions in ecological models^18^,^19^,^20^, palaeoclimate records^21^,^22^, structural behavior^23^, medicine^24^, chemical^25^ and biological systems^26^,^27^.

Critical transitions that occur in many engineering systems result in a huge loss of revenue. One such engineering system is the power generating system where combustion of fossil fuels is the source of power^28^,^29^. Land based gas turbine engines, jet engines used for aviation, and rocket engines fall into this category of combustion dependent power generating systems. In a combustion system, the burning of fuel occurs in an unsteady flow field. The perturbations in the flow causes fluctuations in the heat release rate which results in the generation of sound waves. The sound waves get reflected back to the source, if the entire process occurs in a confinement. These reflected waves modify the heat release rate, forming a feedback loop. A positive feedback could be established when the pressure fluctuations due to the sound waves and the heat release rate fluctuations are favorably phased^30^. The positive feedback can result in the growth of pressure fluctuations and the system can finally attain a state of large amplitude periodic oscillations. The transition to oscillatory behavior occurs as a result of the feedback mechanism that exists between the sound waves and the heat release rate. Hence, combustion systems that exhibit this transition are termed as thermoacoustic systems. The high amplitude periodic oscillations established in the system could cause structural damage, reduce performance of the power generating system or even result in disastrous events such as operational failure of space rockets^31^. Thus, early detection of the transition to self-sustained oscillations is critical in a combustion system.

In general, physical systems display complex dynamics which makes it difficult to predict the transitions or even to describe the nature of the transitions observed in such systems. Therefore, controlled laboratory-scale experiments are necessary to verify the robustness of the early warning measures in physical systems. In this study, we employ ingenious laboratory-scale experiments along with a theoretical model to study the effectiveness of the early warning indicators. This is the first attempt to apply these early warning measures to predict subcritical Hopf bifurcation in a prototypical thermoacoustic system. The experimental set-up consists of a horizontal duct with a heat source located inside the duct. The experimental system could undergo a transition to self-sustained pressure oscillations for suitable choice of its control parameters. In addition to experiments, we use a theoretical model derived from the conservation equations of momentum and energy.

In this study, we identify early warning signals such as increase in variance and conditional heteroskedasticity close to a subcritical Hopf bifurcation. We find that these measures are robust even when the system under consideration is under the influence of external noise. Many physical systems undergo critical transition from a non-oscillatory state to an oscillatory state, which can be described via a subcritical Hopf bifurcation. Hence, our study on the viability of the early warning measures to predict transition in a physical system gains considerable significance.

## Results

### Experimental set-up

**Figure 1:**
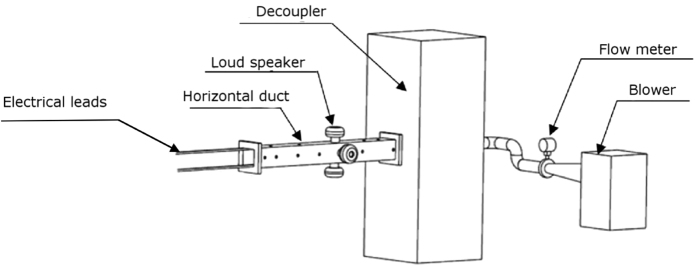
Schematic of the experimental set-up. A blower (Rosemount 3051 SFC) is used to provide the mean flow and a flow meter is used to measure the flow rate. A DC power supply unit is used to heat the wire mesh. We acknowledge Mr. Dileesh M., Junior Technician in the Department of Applied Mechanics, Indian Institute of Technology Madras, for providing the schematic.

The experimental set-up consists of a 1 m long duct of 10 cm × 10 cm square cross-section (Fig. 1). The duct is open to atmosphere at one end. The other end is connected to a rectangular chamber of size much larger than the cross-section dimensions of the duct. This chamber is referred to as decoupler^32^. A blower in the suction mode is used to establish the air flow through the duct. The decoupler isolates the duct from the fluctuations upstream. Thus, the pressure at both the ends of the duct are equal to the ambient pressure. Therefore, the pressure fluctuations at the boundaries are negligible.

An electrically heated wire mesh located in the upstream half of the duct acts as a source of heat for this prototypical system, unlike a flame in practical combustion systems. The voltage drop across the mesh and the current flowing through the mesh are measured to estimate the electrical power supplied to the mesh. We used loudspeakers to provide external fluctuations in experiments when we studied the robustness of early warning measures in the presence of noise.

### Summary of the model

The theoretical model considered in this study captures the feedback between the fluctuating heat release rate and the sound waves (pressure and velocity fluctuations). This feedback could be modified, by varying certain control parameters, resulting in a state shift to large amplitude periodic oscillations. The formulation of the model is detailed in the Methods section. The following set of equations, derived from the conservation equations of momentum and energy, describes the temporal evolution of the system^33^.


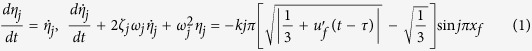


The model described in equation (1) represents a nonlinear self-excited oscillator and it displays a transition from a non-oscillatory state to an oscillatory state via subcritical Hopf bifurcation for suitable change in control parameters. Although the experimental system under study is laminar, aperiodic fluctuations exist in the flow field. These fluctuations in the flow field arise from the blower. The flow noise generated from the blower does not completely decay in the decoupler. These small amplitude fluctuations convect downstream giving rise to small amplitude fluctuations in the flow field. Thus, the base state of the system can be viewed as a laminar flow field superimposed with the low amplitude fluctuations arising from the blower. We chose additive Gaussian white noise^34^ to model these background fluctuations in the theoretical model as it captures the dynamics observed in experiments qualitatively. This qualitative similarity between the experimental results and the theoretical model, when the source of fluctuations is modeled as additive Gaussian white noise, is reported both in the case of a prototypical thermoacoustic system^34^ and in the case of an industrial gas turbine combustor^35^. Once we include the noise, equation (1) becomes:


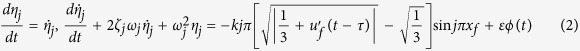


where, *ε* represents strength of the additive Gaussian white noise *ϕ*(*t*). The noise term *ϕ*(*t*) in equation (2) models both background fluctuations in the flow field and the external fluctuations imposed by the loud speaker. The sources of both these fluctuations; the fluctuations due to flow noise from the blower and the fluctuations induced by the loudspeaker; are external to the system. Hence, we use additive noise to model both these fluctuations. The non-dimensional intensity of background fluctuations and external fluctuations is referred to as *β*. The procedure to obtain *β* is detailed in the Methods section. In order to simulate the experimental conditions, we maintained the strength of additive Gaussian white noise *ε* in the model such that the non-dimensional noise intensity *β* is the same as that in the experiments^34^.

### Critical transition associated with a subcritical Hopf bifurcation

**Figure 2:**
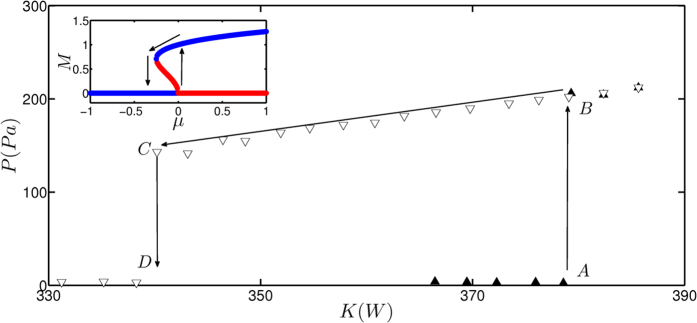
The bifurcation diagram obtained from experiments. The non-oscillatory state loses its stability at point *A* (Hopf point) and the system undergoes a transition from non-oscillatory state to large amplitude oscillatory state as the parameter *K* is increased. The system returns to the non-oscillatory state at point *C* as *K* is reduced. We can observe a bistable region *ABCD*, where the system can remain either in a stable oscillatory state or in a stable non-oscillatory state. The inset shows, the variation of any measure *M* with control parameter *μ* obtained from the normal form equation associated with subcritical Hopf bifurcation. The stable and unstable branches are colored blue and red respectively.

A bifurcation analysis was performed in order to characterize the nature of the transition observed in experiments. The control parameter in this study, the heater power (*K*), was increased in a quasi-steady manner and the time series of unsteady pressure pertaining to each value of the parameter was recorded. When *K* was increased above a threshold value *A*, the median of the peak acoustic pressure (*P*) registered an abrupt increase (see Fig. 2).

In Fig. 2, the abrupt increase in *P* corresponds to a transition of the system from a non-oscillatory to oscillatory state. It is evident from earlier studies that the oscillatory state corresponds to a state of limit cycle oscillations^32^,^34^. Once the system reaches the oscillatory state, the parameter *K* should be reduced to a threshold value further below *A*, to bring the system back to the non-oscillatory state. The threshold values at which the transitions occur are different for the forward path (when *K* is increased) and for the reverse path (when *K* is decreased). This difference in threshold values results in the presence of a bistable regime. The presence of the bistable regime indicates that the transition to the oscillatory state observed in the experimental system is subcritical in nature^36^. The subcritical nature of Hopf bifurcation prompts us to use early warning measures to predict the occurrence of this transition.

In experiments, we have observed a 5% uncertainty in the value of the Hopf point. The uncertainty arises because the experimental conditions such as the mean temperature and the speed of sound are not exactly repeatable. Thus, in a particular experiment, we can expect the onset of oscillations at any *K* within the range of approximately 380 ± 20 W.

### Critical slowing down indicators at the onset of critical transition

We calculated variance, lag-1 autocorrelation and also conditional heteroskedasticity of the time series in search for forewarning signals before the system reaches the state of self-sustained oscillations. Unlike a laboratory experiment where the control parameter is varied in a quasi-static way, the control parameter must be varied continuously as in the case of real systems. This prompted us to perform experiments by varying the control parameter in a continuous manner and capture the time series depicting the continuous transition from non-oscillatory state to oscillatory state. We varied *K* by 2 W in every 20 s and recorded the corresponding time series of acoustic pressure. A plot that depicts the change in K with time is included as Supplementary Fig. S3. The metric based methods suggested by Dakos *et al*.^19^ are adopted to calculate the early warning measures. The time series prior to the transition alone is used for calculating the early warning measures as we require precursors for an impending transition.

**Figure 3:**
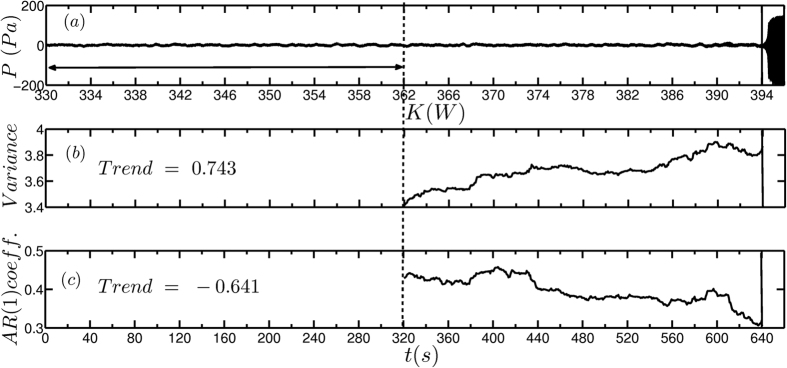
Early warning signals for a critical transition associated with a subcritical Hopf bifurcation using the time series of unsteady pressure obtained from the experiments. (**a**) Time series depicting transition from a stable to an alternate stable state where the heater power *K* is increased by 2 W in every 20 seconds from 330 W to 396 W. The oscillations start to grow at around *t* = 644 s. Plot depicting the change in (**b**) variance and (**c**) lag-1 autocorrelation as the system approaches the critical transition. The lag-1 autocorrelation and variance are calculated using a moving window of half the size of the time series. The black horizontal arrow represents the length of the moving window. The thick solid black line indicates the time stamp (*t* = 640 s) up to which the data is used to calculate the early warning measures while the vertical dotted line indicates the time stamp from which the early warning measures are calculated. We observe a clear increase in variance, well before the transition, whereas the autocorrelation shows a decrease. Although, we have not added external noise to the system, the background fluctuations in the system correspond to a non-dimensional noise intensity *β* = 0.02. A concurrent increase or decrease in the values of a measure (variance or autocorrelation) is identified as trend. We have adopted Mann-Kendall test^37^ to calculate the trend.

In Fig. 3a, the amplitude of the oscillations starts to grow only at around *t*  =  644 s which marks the occurrence of Hopf bifurcation (at *K* = 394 W). The effects of critical slowing down, which can be observed just before the occurrence of the subcritical Hopf bifurcation, will aid in detecting the impending transition. Therefore, we used the time series up to *t* = 640 s to calculate the early warning measures. We can observe a significant increase in the value of variance well before the transition, which clearly serves as an early warning signal (Fig. 3). However, the lag-1 autocorrelation decreases before the transition, rendering it as a less effective early warning measure (Fig. 3).

**Figure 4:**
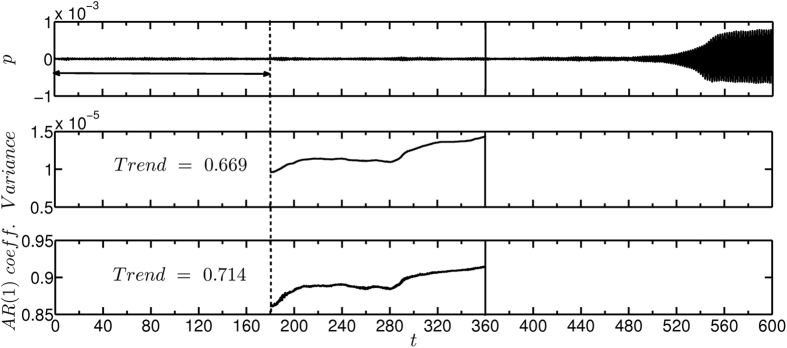
Early warning signals for subcritical Hopf bifurcation using the time series generated from the model (equation (2)). (**a**) Time series of acoustic pressure depicting the transition from a stable to an alternative stable state. The control parameter *k* is increased as a function of time, given by *k* = 1.667 × 10^−3^ *t,* from 0 to 1. The parameter reaches the Hopf point (*k* = 0.62) at *t* = 372. Plot depicting the change in (**b**) variance and (**c**) lag-1 autocorrelation as the system approaches the critical transition. We observe increase in variance and autocorrelation well before the transition. We maintained the value of *ε* such that the non-dimensional noise intensity is 0.02 to match the experimental conditions. Note that the parameter *k* and the acoustic pressure that we calculate from the model are non-dimensional.

We extended our search for effective early warning measures for predicting a subcritical Hopf bifurcation also in the theoretical model given by equation (2). We varied the non-dimensional control parameter (*k*) as a function of time, given by *k* = 1.667 × 10^−3^ *t,* and obtained the corresponding time series of unsteady pressure. Here also, we observed a transition to a state of limit cycle oscillations^38^. The lag-1 autocorrelation and variance were calculated using the same procedure that we followed for the time series obtained from experiments. In the model, we observe that the values of variance and lag-1 autocorrelation increase well before the transition (Fig. 4). Lag-1 autocorrelation shows a decreasing trend in experiments while we observe an increasing trend in the model. In experiments, the system has background aperiodic fluctuations, though we have not added any external noise in the system. The presence of fluctuations makes autocorrelation a less effective precursor as it is established that autocorrelation can increase or decrease in the presence of fluctuations^39^. In order to use autocorrelation as a precursor, we need to have multiple realizations of the transition, which may not be feasible when it is applied as an early warning measure in a real time system^36^. The observations from the model and experiments make it clear that variance is a more robust early warning measure as compared to lag-1 autocorrelation. Our observation of variance being a more robust early warning measure than lag-1 autocorrelation is completely in conformity with Ghanavati *et al*.^40^.

**Figure 5:**
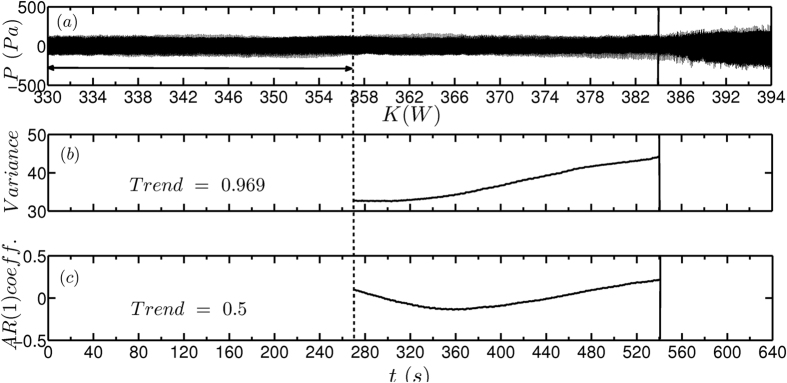
Early warning signals for subcritical Hopf bifurcation in the presence of external fluctuations using the time series of unsteady pressure acquired from the experiments. (**a**) Time series of acoustic pressure depicting transition from a stable to an alternate stable state where the heater power *K* is varied by 2 W in every 20 s from 330 W to 394 W. Plot depicting the change in (**b**) variance and (**c**) lag-1 autocorrelation as the system approaches the critical transition. We observe a rise in variance and autocorrelation before the transition. We have added external noise to the system such that the non-dimensional noise intensity is *β* = 0.2.

The robustness of the early warning indicators must be tested in the presence of fluctuations as practical combustion systems work in a turbulent environment. Therefore, we added external noise of non-dimensional intensity *β* = 0.2 in the system using loudspeakers^34^. Here also, we changed the control parameter by 2 W in every 20 s and recorded the time series of unsteady pressure. We observe that lag-1 autocorrelation shows an increasing trend close to the transition and variance shows a concurrent rise indicating the proximity to a critical transition (Fig. 5). Our experimental results prove that early warning indicators perform very well even in the presence of external fluctuations of intensity one order higher than that of the background fluctuations.

**Figure 6:**
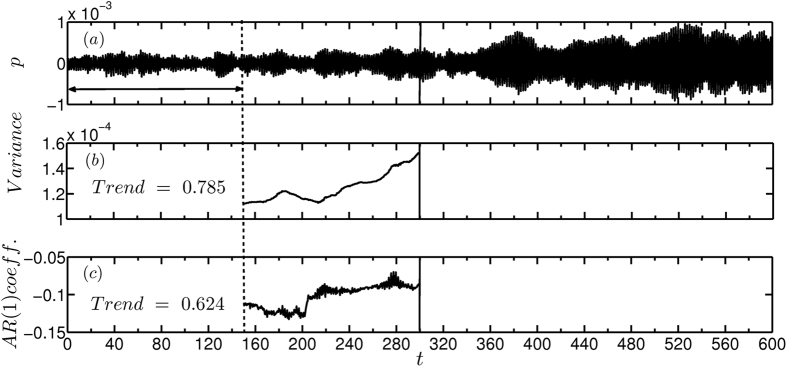
Early warning signals for subcritical Hopf bifurcation in the presence of high intensity additive noise using the time series generated from the model (equation (2)). (**a**) Time series of unsteady pressure depicting the transition from a stable to an alternate stable state. The control parameter *k* is increased as a function of time, given by *k* = 1.667 × 10^−3^ *t,* from 0 to 1. Plot depicting the change in (**b**) variance and (**c**) lag-1 autocorrelation as the system approaches the critical transition. We observe a clear increase in variance and autocorrelation well before the transition. We maintained the value of *ε* such that the non-dimensional noise intensity is 0.2 to match the experimental conditions.

We further tested the robustness of early warning signals using the model (equation (2)). In the model, we applied Gaussian white noise such that the non-dimensional intensity is 0.2 to match the experimental conditions. The value of the Hopf point (*k* = 0.62) does not change since the noise in the model is additive in nature^41^. The parameter *k* reaches the value of Hopf point only at *t* = 372. We can notice in Fig. 6(a) that the amplitude starts to grow much before the Hopf point is reached. Therefore, the transition to limit cycle presented in Fig. 6(a) represents noise induced tipping in the system. In this case also, we observed a trend similar to that observed in experiments, where variance and lag-1 autocorrelation increase well before the transition (Fig. 6). Our results show that the early warning measures perform well in forewarning critical transitions irrespective of the presence of background and external fluctuations.

We then proceed to calculate conditional heteroskedasticity which was introduced as an early warning indicator by Seekell *et al*.^18^. Conditional heteroskedasticity is indicated by the persistence in the conditional variance of the error terms. We followed the procedure suggested by Seekel *et al*.^18^ to estimate conditional heteroskedasticity (refer Methods section for details of the procedure). In this procedure, the time series is modeled as an autoregressive process and the residuals are obtained. We then estimate the conditional heteroskedasticity by examining the persistence in the conditional variance of the residuals. We adopt conditional heteroskedasticity as an additional measure along with variance and autocorrelation to early warn the transition.

**Figure 7:**
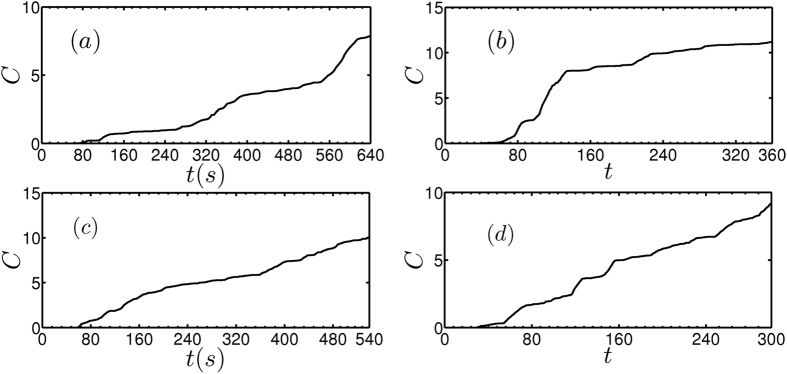
The cumulative number of significant Lagrange multiplier test (*C*) applied to the time series obtained from the experiments and the model. In (**a**) to (**d**) the time series used are same as in Figs 3, 4, 5, 6, respectively. The cumulative *C* increases close to the transition indicating that significant number of tests shows conditional heteroskedasticity.

We consider the time series data used in Figs 3, 4, 5, 6 to calculate the conditional heteroskedasticity. We identified the significant number of tests where conditional heteroskedasticity is observed. We find that the cumulative number of significant tests (denoted by *C*) for conditional heteroskedasticity, applied to the time series, increases as the critical transition is approached (Fig. 7). The increase in *C* for all the cases considered in this study ascertains regime shift in the system^18^.

## Discussion

We find that the critical slowing down indicators, e.g., variance and conditional heteroskedasticity, as early warning signals are able to predict catastrophic transitions in a physical system. These measures are robust even in the presence of high intensity noise. In the model, lag-1 autocorrelation shows an increasing trend in the presence of low and high intensity noise. However, in experiments, lag-1 autocorrelation shows a decreasing trend (in the presence of background fluctuations) as the critical point is approached, though it shows an increasing trend in the presence of external noise imposed by a loud speaker. The lack of robustness of autocorrelation as a precursor is due to the fact that we are using a single sample path or a single realization^36^.

This is the first experimental study where we establish the effectiveness of critical slowing down indicators in early warning critical transition in a thermoacoustic system. Our findings are highly pertinent as many real systems exhibit critical transitions. By implementing the early warning measures in systems such as the one considered in the present study, we can prevent critical transitions in real systems. We wish to emphasize that the early warning indicators exploit the phenomenon of critical slowing down which happens near any bifurcation. Therefore, any transition which can be viewed as a bifurcation can be predicted using the early warning measures.

There are certain limitations to the approach we have adopted in this study. The early warning indicators considered in the current study may fail to detect the regime shifts that occur as a result of rapid change in the control parameter^42^. Apart from this, the early warning measures might not detect transitions induced by a finite amplitude perturbation in the system. The perturbation can occur at any arbitrary instant of time and can take the system to the basin of attraction of the stable limit cycle. Tipping in the system as a result of perturbations is not preceded by critical slowing down. Hence, the early warning indicators discussed in this study might not detect this transition. We provide an example from our experiments and mathematical model where early warning measures fail to detect a transition induced by a sudden finite amplitude perturbation (see Supplementary material).

## Methods

### Data acquisition

We use piezoelectric transducers (PCB piezotronics, PCB103B02) to record the variation in acoustic pressure. The sensitivity of the pressure transducer is 217.5 mV/kPa and the resolution is 0.2 Pa. The output from the pressure transducer is acquired using a data acquisition system (PCI 6221) through a signal conditioner. The transducer is mounted 325 mm from the end open to the atmosphere. We add external noise to the system through loud speakers (Ahuja AU 60) mounted on the duct 625 mm from the end open to the atmosphere^35^. The white noise signal is created in LabVIEW software and then input to the loud speakers through an amplifier. A DC power supply (TDK-Lambda, GEN 8-400) of range 0–8 V and 0–400 A is used to heat the wire mesh. The wire mesh is made from mild steel. The uncertainty in the heater power is 0.4 W. The accuracy of the flow meter is ± 0.1%. The decoupler has dimensions 1200 mm × 450 mm × 450 mm.

### Theoretical model

The model is derived from the conservation equations of momentum and energy^33^. In the model, we consider a horizontal duct with a concentrated heat source located inside the duct. An average value of temperature in the duct is adopted to model the sound propagation in the duct. We can write the conservation equations of momentum and energy (in one-dimension) as follows^43^:


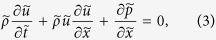



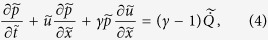


where, *γ* is the ratio of specific heats of air. The quantities with tilde are dimensional. The variables pressure (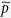), density (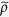), velocity (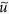) and heat release rate (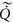) are decomposed into mean and fluctuating quantities. For example, density is expressed as 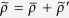. The fluctuating quantities are much small compared to the mean. For instance, the amplitude of the pressure fluctuations is less than 1% of the mean pressure in Rijke tube. It implies that the propagation of sound waves in the duct can be modeled as a linear phenomenon. Therefore, the linear terms involving the fluctuating variables alone are retained and higher order terms are neglected. In addition, the low Mach number approximation^44^ is adopted to obtain the following equations:


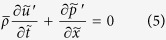



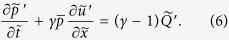


The fluctuating heat release rate per unit volume (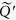) is given by the following relation^45^:


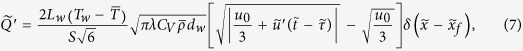


where, *λ* is the heat conductivity of air, *C*_*V*_ is the specific heat at constant volume, *L*_*W*_ is the effective wire length, 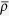 and 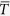 are the mean density and temperature of the flow respectively, *T*_*W*_ is the temperature of the wire, *d*_*W*_ is the wire diameter and *S* is the cross-sectional area of the duct. The unsteady heat release rate is a nonlinear function of the velocity fluctuations at the heater location. The heat release rate responds to the velocity fluctuations after a delay (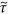) which is incorporated in equation (7). The physical reason for this delay is the presence of the thermal and the hydrodynamic boundary layers around the wire. Due to the presence of the boundary layer, the heat release rate from the wire responds to the velocity fluctuations after a delay. The following expression can be used to estimate the non-dimensional time delay from experiments^46^.


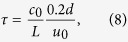


where *c*_0_ is the speed of sound, *L* is the length of the duct, *u*_0_ is the mean speed of the flow and *d* is the diameter of the cylindrical heating wire.

Moreover, a similar delay is expected in our experimental set-up, as we have a mesh type heater (which can be viewed as an array of cylindrical wires). In a practical combustion system, this delay can be attributed to factors such as the convective time scale (time taken by the fuel air mixture to travel the distance between the fuel injection point and the flame location), evaporation time scale, mixing time scale and reaction time scale.

The location of the heat source inside the duct is denoted by 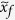. Although the sound propagation is modeled as a linear phenomenon, the interaction between heat release rate and the sound waves is nonlinear. Therefore, equations (5) and (6) still represent a nonlinear system of equations as the expression for heat release rate is nonlinear^33^. Equations (5) and (6) are then expressed in terms of non-dimensional quantities by adopting the following scales for non-dimensionalization.


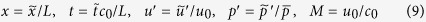


The quantities with tilde are dimensional. *M* is the mean flow Mach number and 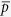 is the steady state pressure. The following equations are obtained after non-dimensionalization.


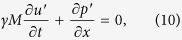



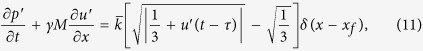


where, 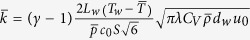.

The variables that govern the heat conduction from the wire are encapsulated into a single non-dimensional parameter denoted by 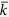. In the analyses using the model, 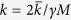, is used as the control parameter analogous to the heater power in experiments. The non-dimensional acoustic pressure and acoustic velocity are represented by 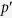 and 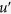. The acoustic pressure (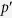) and the acoustic velocity (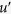) are expressed in terms of the natural spatial modes of the duct as follows^33^:


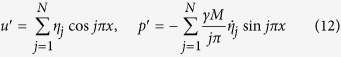


Here 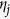 and 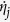 represent the time varying coefficients of *j*^*th*^ natural mode. The expansions for 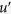 and 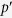 from equation (12) are substituted into equations (10) and (11) to yield equation (1).

The damping in the system is modeled using the term 
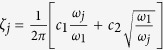
, where *c*_1_ and *c*_2_ are the damping coefficients^32^,^47^. *ω*_*j*_ = *jπ* represents the non-dimensional angular frequency of the *j*^*th*^ mode. The non-dimensional frequency *ω*_*j*_ is obtained by non-dimensionalizing the angular frequency of the *j*^*th*^ natural mode of a duct open at both ends, 
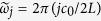
. 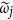 is non-dimensionalized with *c*_0_/*L*, where *c*_0_/*L* is the travel time of the sound waves in the duct. The value of the parameters used for computation are *N* (number of modes) = 10, *dt* (step size) = 0.01, *ω*_*j*_ = *jπ*, *c*_1_ = 0.1, *c*_2_ = 0.06, *x*_*f*_ = 0.25 and *τ* = 0.2.

The amplitude of fluctuations or the noise level in the system (both in experiments and in the theoretical model) is estimated by measuring the rms amplitude of the acoustic pressure when the system is in the non-oscillatory state. The noise amplitude (*I*) is non-dimensionalized by the amplitude of limit cycle attained at the Hopf point in the absence of external noise (*P*_*H*_).


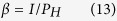


Here, *β* is the non-dimensional noise intensity.

### Derivation of early warning signals

We performed the statistical analyses using the “Early Warning Signals Tool Box” (http://www.early-warning-signals.org/). We used the time series up to the impending critical transition to calculate the early warning indicators. For variance and autocorrelation, we calculated the temporal trend by estimating the nonparametric Kendall rank correlation (*τ*_*k*_)^48^ Kendall’s *τ*_*k*_ is a statistical tool used to measure the association between two measured quantities. Lag-1 autocorrelation is determined by the autocorrelation function (ACF) given below:


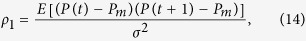


where, *P*(*t*) is the value of the state variable at time *t*, and *P*_*m*_ and *σ*^2^ are the mean and variance of *P*(*t*) within the time frame considered. Variance is calculated as:


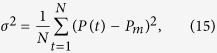


where 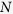 is the number of observations.

We used Lagrange multiplier test to calculate conditional heteroskedasticity^49^. For this, we fit autoregressive model of selected order to the time series.


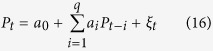


The order is selected according to Akaike information criterion^50^ which is a measure of the relative goodness of the fit. After fitting of model to the time series, we calculated squared residuals (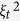) and regressed them one time step


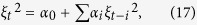


where *α*_0_ and *α*_*i*_ represent the regression coefficients. The residuals give the error variance relationship which shows the properties of conditional heteroskedasticity. We conducted chi square test to compare the values of *R*^2^ (Lagrange multiplier test statistic) to a 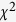 distribution so as to identify the number of significant tests where conditional heteroskedasticity is observed.

## Additional Information

**How to cite this article**: Gopalakrishnan, E. A. *et al*. Early warning signals for critical transitions in a thermoacoustic system. *Sci. Rep.* **6**, 35310; doi: 10.1038/srep35310 (2016).

## Supplementary Material

Supplementary Information
