# Supplementary material for: Early warning signals for critical transitions in a thermoacoustic system
Source: Sci Rep. 2016 Oct 21;6:35310. doi: 10.1038/srep35310 (PMC5073343; doi:10.1038/srep35310)
Supplement: Supplementary Information [file srep35310-s1.pdf]

Supplementary material

Early warning signals for critical transitions in a thermoacoustic system

Gopalakrishnan E. A.[1], Yogita Sharma[2], Tony John[1], Partha Sharathi Dutta[2], and Sujith R. I.[1]

[1]Department of Aerospace Engineering, Indian Institute of Technology Madras, India - 600036.
[2]Department of Mathematics, Indian Institute of Technology Ropar, India - 140001.

A. Early warning measures close to a transition induced by a finite amplitude perturbation

In this document, we provide an example from our experiments and mathematical model where early warning measures fail to detect a transition induced by a finite amplitude perturbation.

In experiments, the parameters are chosen such that the system is initially in the non-oscillatory state (marked by aperiodic fluctuations due to background noise) in the bistable region. Then, the system is perturbed by a finite amplitude disturbance that transitions it to the oscillatory state. The time series corresponding to this transition (Supplementary Fig. S1) is used to calculate the early warning measures. We can observe from Supplementary Fig. S1(b) that variance fails to early warn the transition.

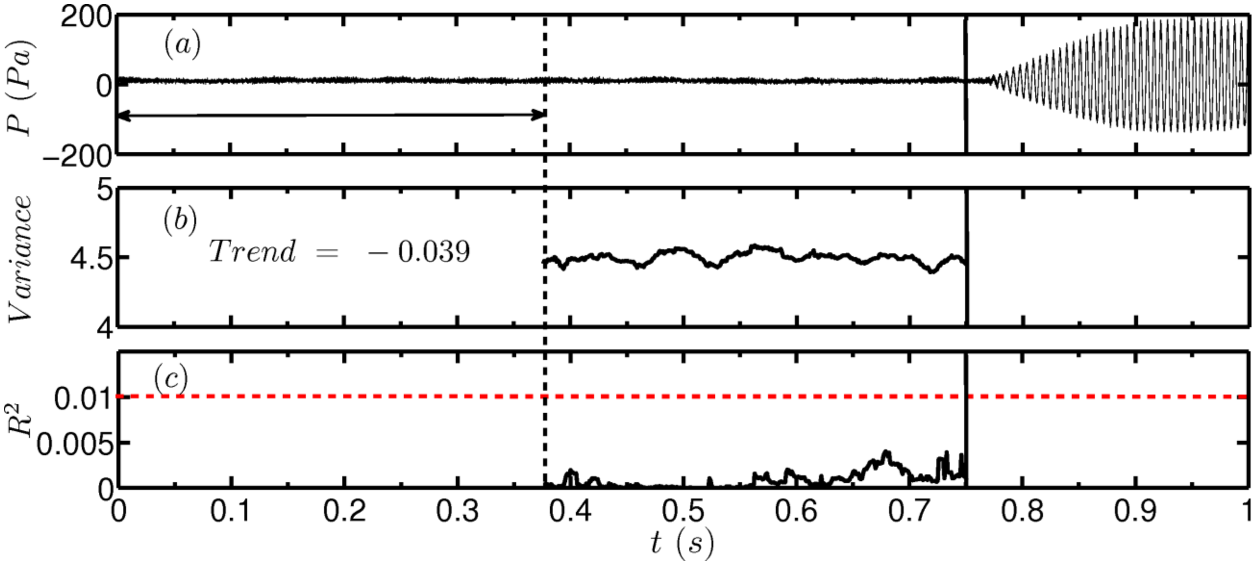

Supplementary Figure S1. **Early warning indicators close to a transition induced by a finite amplitude perturbation in experiments**. (a) Time series of unsteady pressure depicting the transition from aperiodic to large amplitude periodic oscillations acquired from experiments. The system is given a finite amplitude disturbance at t = 0.77 s which transitions the system to the oscillatory state. Plot depicting the change in (b) variance and (c) Lagrange multiplier test statistic prior to the transition. We can observe that variance does not provide any indication of the impending transition. We also do not observe significant conditional heteroskedasticity prior to the transition. The red line denotes the threshold above which the value of the statistic is significant.

We also calculated conditional heteroskedasticity for the time series shown in Supplementary Fig. S1(a). The variation of the Lagrange multiplier test statistic (R^2) with time is shown in Supplementary Fig. S1(c).

A significant number of tests above the threshold (red line) is an indication of conditional heteroskedasticity. The threshold value is chosen such that we can confirm that the time series is conditional heteroskedastic with 95 % confidence level.

The number of significant tests tends to increase consistently prior to a transition associated with a bifurcation. We can notice in Supplementary Fig. S1(c) that the values of the statistic are below the threshold even close to the transition. Therefore, conditional heteroskedasticity does not detect the transition induced by a finite amplitude perturbation in the system.

Further, we performed numerical experiments to illustrate the ineffectiveness of variance and conditional heteroskedasticity in early warning a transition induced by a sudden finite amplitude perturbation (see Supplementary Fig. S2). The measures (variance and conditional heteroskedasticity) do not early warn the transition because it is not preceded by critical slowing down.

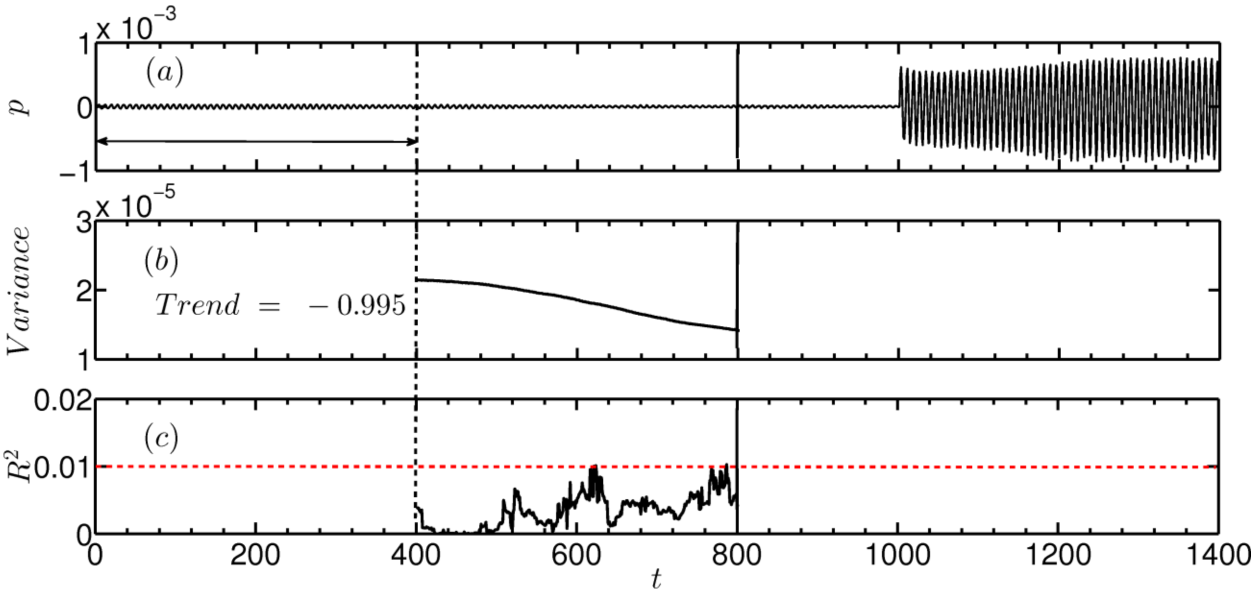

Supplementary Figure S2. **Early warning indicators close to a transition induced by a finite amplitude perturbation in the model**. (a) Time series of unsteady pressure depicting the transition from aperiodic to large amplitude periodic oscillations. The system is given a finite amplitude disturbance at t = 1000 which transitions the system to the oscillatory state. Plot depicting the change in (b) variance and (c) Lagrange multiplier test statistic prior to the transition. Variance fails to early warn the transition as it shows a decrease prior to the transition. We do not observe significant conditional heteroskedasticity prior to the transition.

B. Variation of heater power in experiments

The variation of heater power (*K*) with time in experiments is shown in Supplementary Fig. S3. Initially, *K* is maintained at a value of 330W. The value of *K* is changed after every 20 s. The heater power is changed in this manner to obtain the time series presented in Figs. 3(a) and 5(a) of the manuscript.

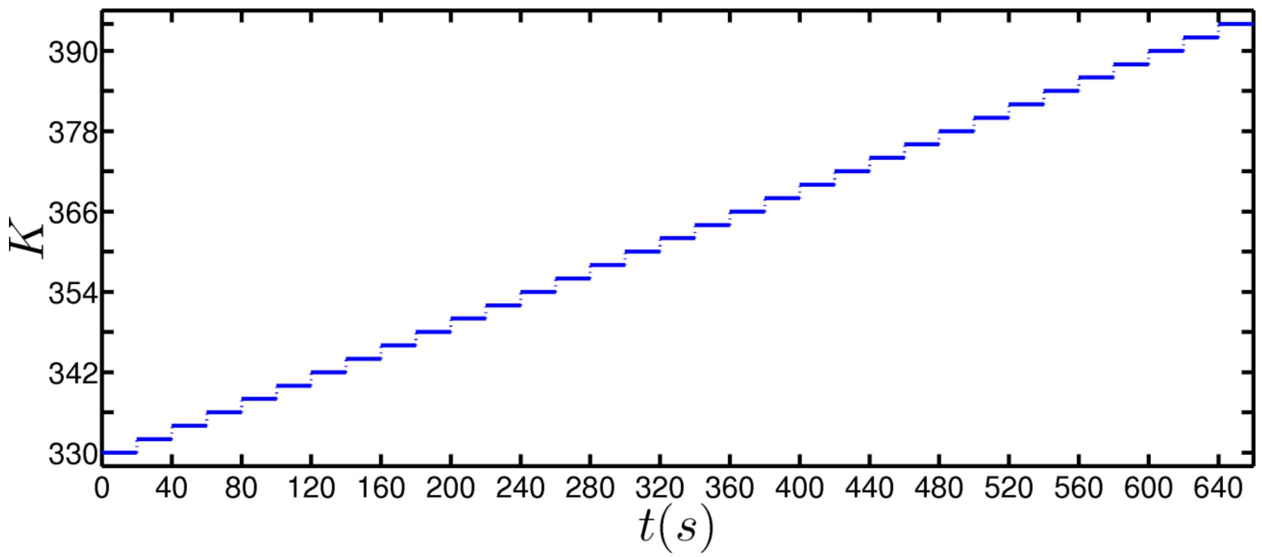

Supplementary Figure S3. **The variation of heater power in experiments.** The value of K is initially maintained at 330W for 20s. We increment the value of K by 2W after every 20 s.
